# Transcriptional Activation of the Human CD2AP Promoter by E2F1

**DOI:** 10.1371/journal.pone.0042774

**Published:** 2012-08-03

**Authors:** Li Zou, Hua-Guo Xu, Wei Ren, Rui Jin, Yi Wang, Guo-Ping Zhou

**Affiliations:** 1 Department of Pediatrics, The First Affiliated Hospital, Nanjing Medical University, Nanjing, Jiangsu Province, China; 2 Department of Clinical Laboratory, The First Affiliated Hospital, Nanjing Medical University, Nanjing, Jiangsu Province, China; Kyushu Institute of Technology, Japan

## Abstract

CD2-associated protein (CD2AP) is an adaptor molecule involved in T cell receptor signaling and podocyte homeostasis. CD2AP-deficient mice develop nephritic syndrome and renal failure caused by glomerulosclerosis. Transcription factor E2F1 is a key regulator of cell proliferation and apoptosis. Here we report that E2F1 up-regulates the human CD2AP promoter and further increases the mRNA and protein levels of the human CD2AP in human embryonic kidney (HEK) 293 cells. By semi-quantitative RT-PCR and Western blot analysis we demonstrate that ectopic expression of E2F1 elevates the mRNA and protein levels of CD2AP. Consistently, transient transfection assays prove that overexpression of E2F1 transactivates the CD2AP promoter while knocking-down of endogenous E2F1 by a shRNA strategy results in reduction of the CD2AP promoter activity. Toward understanding the underlying mechanism of this regulation, we performed chromatin immunoprecipitation and mutations of the putative Sp1 binding sites, demonstrating that E2F1 can bind to Sp1 binding site and overexpression of E2F1 is capable of increasing the binding of E2F1 and decreasing the binding of Sp1 to Sp1 binding sites.

## Introduction

CD2-associated protein (CD2AP), an 80-kDa protein, was originally cloned as a protein that interacts with the cytoplasmic domain of CD2, a T lymphocyte and natural killer-specific membrane protein [1]. Although originally cloned from a lymphocyte library, CD2AP is highly expressed in podocytes and widely expressed in all tissues except brain [1]. CD2AP is involved in cytoskeletal remodeling [2], [3], cell survival [4], [5], and endocytosis [6]–[8]. Recently, CD2AP was found to be related to glomerulosclerosis. CD2AP knockout mice were generated and found to develop a rapid onset nephrotic syndrome and renal failure at 3 weeks of age. All of the mice died at 6 to10 weeks of age [3], [9]–[11]. CD2AP is considered to play an important role in the maintenance of the glomerular slit diaphragm [12]. It has been demonstrated now that CD2AP is widely involved in several signaling pathway. Schiffer M, et al. reported that CD2AP was required for early activation of anti-apoptotic phosphatidylinositol 3-kinase (PI3K)/AKT and extracellular signal-regulated kinase 1/2 by TGF-beta [5]. Another study by Lu C et al. proved that EGF/AP-1/CD2AP axis functions as a survival signal transduction cascade and suppress angiotensin II-induced apoptosis in renal tubular epithelial cells [13].

Members of the E2F transcription factor family are important regulators of cell proliferation, differentiation and apoptosis. E2Fs control the cell cycle by regulating the expression of a number of genes, whose products are required for the S-phase entry and cell cycle progression [14]. Usually, individual E2F proteins are classified into three categories based on their transcriptional properties and their interaction potential with pocket proteins (Rb, p107, p130). E2F1, 2, and 3 are potent transcriptional activators that interact directly with RB. E2F4 and 5, on the other hand, are weak activators and appear to be able to interact with the three pocket proteins [15]. E2F6, 7, and 8 do not associate with pocket proteins and are believed to repress transcription [16], [17]. The E2F1 factor, the most characteristic member of the E2F transcription factor family, regulates the expression of genes involved in the G1/S transition and DNA synthesis [18]–[20]. E2F1 is transcriptional activator as usual, which binds to DNA and regulates the expression of genes involved in cell cycle progression. However, some studies have demonstrated that E2F1 can also act as transcriptional repressor [21], [22]. The classic E2F binding sequence is 5′-TTTSSCGC-3′(S = C/G), whereas it has been suggested that E2F1 can also bind to Sp1 binding sites and further regulate the downstream target genes [23], [24].

Previously, we have confirmed that transcription factor Sp1 increased the human CD2AP promoter activity through binding to Sp1 binding sites in several cell lines [25]–[27]. In this study, we demonstrate that E2F1 plays an important role in up-regulating CD2AP transcription activity through Sp1 binding site in HEK293 cells.

## Materials and Methods

### Cell culture

HEK293 cells were obtained from the American Type Culture Collection (ATCC). HEK293 cells were maintained in Dulbecco's modified Eagle's medium (DMEM) containing 10% heat inactivated fetal bovine serum (FBS), supplemented with penicillin (100 unit/ml) and streptomycin (100 µg/ml). HEK293 Cells were incubated at 37°C with 100% humidity in 5% CO2 and passaged using standard cell culture techniques.

### Plasmids, transfection, and RNAi

Promoter-luciferase reporter constructs of human CD2AP gene were performed as described previously [25]. The plasmids pcDNA3-E2F1, pcDNA3-E2F1 (E132), pU6-E2F1 shRNA and control vector pU6 were gifts from Dr. W. Douglas Cress [28]. Mutations of the Sp1 binding sites of the human CD2AP promoter were performed using QuikChange Site-Directed Mutagenesis Kit (Stratagene) from the wild-type promoter region. Oligonucleotides with site-specific mutations at the critical nucleotides necessary for transcription factor are listed in Table 1. The mutations were confirmed by sequencing. The expression plasmids pcDNA3-E2F1, pcDNA3-E2F1 (E132) and pcDNA3-empty vector were purified and cotransfected by using Lipofectamine™ 2000 (Invitrogen) and then incubated for 24 h. For RNAi assay, HEK293 cells were transfected with pU6-E2F1 shRNA or control vector and cultured for 48 h before cells were harvested to examine the effectiveness of RNA interference.

**Table 1:** Sequences of oligonucleotides used in site-directed mutagenesis.

Names of plasmids	Sequence
pGL3-0.6k/mA	CCCGAACGCGACGGG**ATT**GGGTGGGGCG
pGL3-0.6k/mB	GGGGCGGGGTGGG**ATT**GGGAGAACGAG
pGL3-0.6k/mC	AGAGGCGAGGG**ATT**GGCTCCGAGGCTAG
pGL3-0.6k/mD	GCGGTCGGGC**ATT**CGGGGTAGGGCCC

Sequences listed above are from 5′ to 3′. Mutations are shown in bold above the table.

### Dual-Luciferase Reporter Assays

Cells were seeded to 96-well plates 24 h before transfection. E2F1 expression plasmid (50–200 ng) or empty vector was individually cotransfected into HEK293 cells, together with appropriate CD2AP promoter reporter plasmids (50 ng) using Lipofectamine™ 2000 (Invitrogen) according to method which was described previously [29]. For RNAi assay, pU6-E2F1 shRNA or control vector was individually, with appropriate CD2AP promoter reporter plasmids, cotransfected into HEK293 cells. The pRL-TK plasmid (Promega; 2 ng/sample) containing the Renilla luciferase gene driven by the herpes simplex virus thymidine kinase promoter was cotransfected with the constructs, and the luciferase activity was normalized. Dual-Luciferase Reporter Assays was performed as described previously [30]. Experiments were performed in triplicate.

### Semiquantitative RT-PCR

For semiquantitative RT-PCR, HEK293 cells were first transfected with E2F1 expression plasmid or empty vector pcDNA3 and were cultured for additional 24 h. Contemporary, cells were transfected with shRNA or empty vector U6 and were cultured for additional 48 h. Total cellular RNA was extracted with Trizol reagent according to manufacturer's protocol. The RNA was reverse transcribed by TaKaRa One-step RNA PCR Kit (AMV) (TaKaRa BIOINC., Japan) using the random primer. The resulting cDNA was subjected to PCR analysis using gene specific primers for human CD2AP: forward, 5′- AACTCATGAAGCCCAGGACGA-3′, reverse, 5′- CTGATCCAGATGCAGTTTCACTCAC-3′; GAPDH: forward, 5′-TACTAGCGGTTTTACGGGCG-3′, reverse, 5′-TCGAACAGGAGGAGCAGAGAGCGA-3′. The PCR amplification was performed by incubating the samples at 94°C for 5 min, followed by 35 cycles of 30 s at 94°C, 30 s at 63.2°C, 30 s at 72°C; and a final extension step for 10 min at 72°C. The amplified products were separated by gel electrophoresis on a 2% agarose gel.

### Western blot analysis

For Western blotting, HEK293 cells were transfected with E2F1 expression plasmid, shRNA or empty vector and were cultured for additional 72 h. The cells were lysed and centrifuged. And then the supernatants were collected. Equal amounts of protein were resolved by 10 or 4–15% SDS-PAGE and transferred onto polyvinylidene difluoride membrane using standard procedures. After blocking in TBST containing 5% dry milk, incubation with primary Ab (1∶200) (Santa Cruz, CA) and then with HRP-conjugated secondary Ab (1∶2500) was performed. Subsequently, membranes were washed and developed with ECL reagent (Pierce, USA). GAPDH were used as loading controls and was detected with mouse monoclonal anti-GAPDH antibody (Santa Cruz, CA).

### Chromotin immunoprecipitation assay (ChIP)

ChIP was performed following the protocol provided in ChIP-IT kit (Active Motif, Carlsbad, CA). Cells were cultured for additional 24 h after them were transfected with E2F1 expression plasmid or empty vector pcDNA. Then, two 100 cm2 dishes of 80∼90%-confluent HEK293 cells were treated with 1% formaldehyde in PBS for 10 min at room temperature. The formaldehyde was inactivated by the addition of 0.125 M glycine in PBS to the cells for 5 min at room temperature. The cells were then washed in ice-cold PBS and then lysed with lysis buffer containing 1% SDS. Sonication of cross-linked chromatin was performed at 200 watt with five rounds of 20 s pulses so that chromatin fragments thus obtained ranged from 200 to 1000 bp in size. Subsequently, anti-E2F1 (C-20) (Santa Cruz Biotechnology),anti-Sp1(Santa Cruz Biotechnology) or IgG control(Active Motif) were added for immunoprecipitating the chromatin. For each immunoprecipitation, 2 µg of the appropriate antibody was incubated with a precleared chromatin aliquot overnight at 4°C. The immunoprecipitates were then pelleted, washed, and the antibody/protein/DNA complex was eluted the beads by resuspension of the washed pellet in 1M NaHCO3 and 1% SDS for 30 min. Following immunoprecipitation and elution, the eluent was added 1 ul RNase A and heated to 65°C for 6 h to remove RNA and reverse the cross-link. Protein were removed by adding 2 ul Proteinase K and incubated at 42°C for 2 h. Then DNA samples were purified using mini-columns provided with the kit. The purified DNA was amplified by the promoter-specific primers (ChIP-F, 5′- ACCTGGCAGCAGCCGTGG-3′ and ChIP-R, 5′-AGGCAAAGGCGGCGACAG-3′), and PCR was performed under the following conditions: 1 cycle at 94°C for 5 min; 36 cycles of 1 min at 94°C, 1 min at 62°C, 30 s at 72°C; and a final extension step for 10 min at 72°C. Input of every sample was used as an internal control. PCR products were separated by gel electrophoresis on 2% agarose gel and visualized.

## Results and Discussion

### E2F1 binds to the human CD2AP promoter in vivo

**Figure 1:**
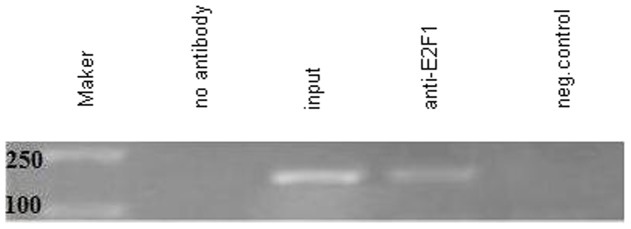
Chromatin immunopreciptation analysis. E2F1 binds to the CD2AP promoter in vivo. HEK293 cells were transfected with pcDNA3 or pcDNA3-E2F1 expression vector. 24 h post-transfection, cells were harvested and subjected to ChIP assay. ChIP products were amplified by PCR reaction. Cross-linked chromatin samples from pcDNA3-transfected HEK293 cells, pcDNA3-E2F1-transfected HEK293 cells were respectively, immunoprecipitated with anti-E2F1, and normal rabbit IgG (anti-IgG). Purified DNA was analyzed by PCR using primers as described in Materials and methods. A sample representing 10% of the chromatin used as a starting material for the immunoprecipitation is also shown (input). The amplified PCR fragments were analyzed on 2.0% agarose gel. Each experiment was performed twice with similar results, and one of them is shown.

Sequence analysis by using the MatInspector algorithm and the UCSC browser revealed that CD2AP minimal promoter positively regulated by Sp1 does not contain any E2F binding sites. Some studies have demonstrated that E2F1 can bind to Sp1 binding sites and further regulate the downstream target genes. Therefore we speculated that E2F1 could regulate the CD2AP promoter activity by Sp1 binding site. Firstly, we performed a chromatin immunoprecipitation assay in untreated HEK293 cells to identify whether E2F1 binds to the CD2AP promoter. The chromatin was immunoprecipitated with anti-IgG and anti-E2F1 antibodies and the DNA precipitated in the complexes was subjected to PCR amplification with primers flanking the region containing four Sp1 binding sites. As shown in Figure 1, an enrichment of the CD2AP promoter was detected using anti-E2F1 antibody in HEK293 cells. No signal was observed using a negative control antibody (normal rabbit IgG). These results indicate that E2F1 can bind to the promoter region of human CD2AP gene in HEK293 cells.

### E2F1 induces CD2AP expression

**Figure 2:**
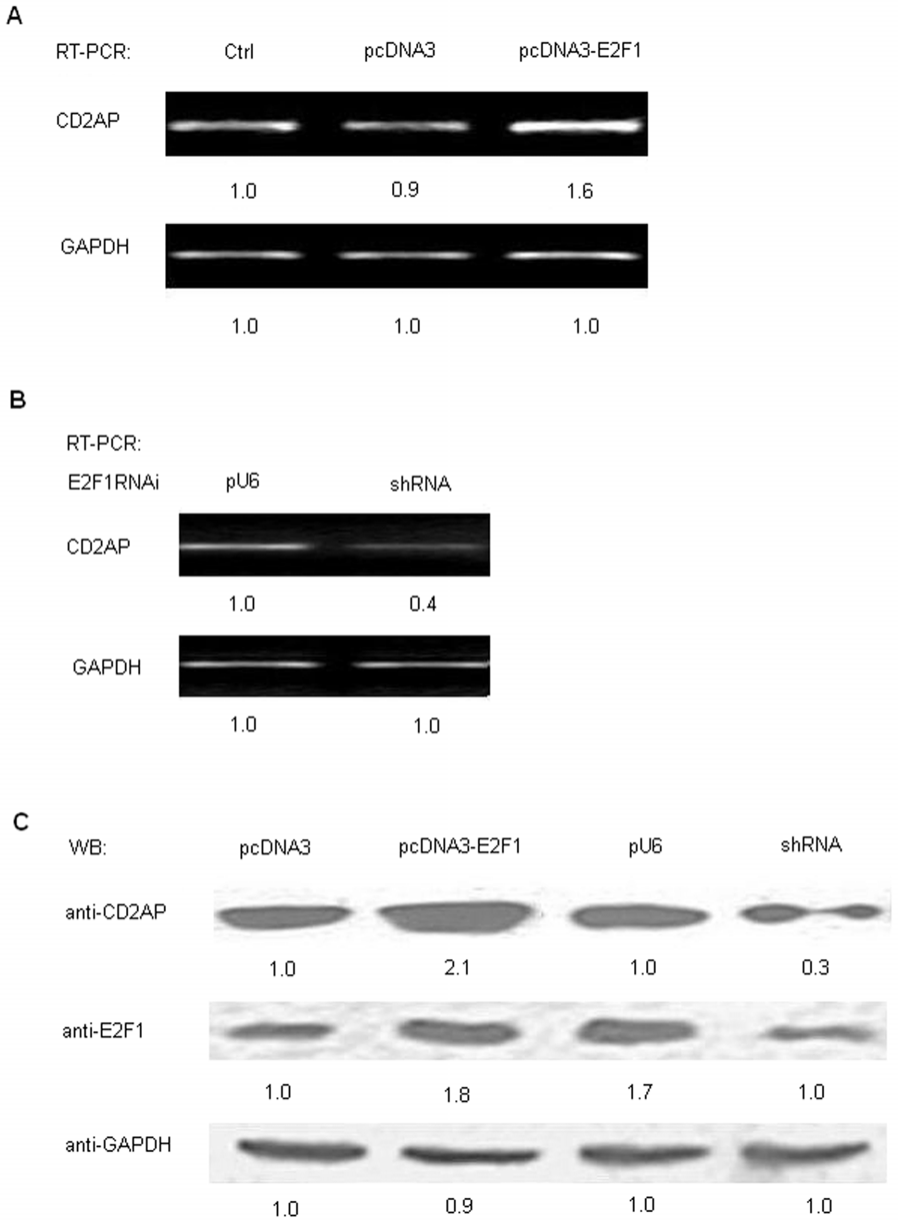
E2F1 up-regulates the expression of CD2AP. Overexpression of E2F1 leads to increased expression of the CD2AP. (A) HEK293 cells were transiently transfected with the pcDNA3 and pcDNA3-E2F1 expression plasmid. 24h post-transfection, cells were harvested for RNA isolation and equal amounts of total RNA were reverse transcribed and amplified by PCR. GAPDH was performed simultaneously as control. (B) HEK293 cells were transiently transfected with the pU6 or pU6-E2F1 shRNA plasmid. Then cells were treated under the same steps as described in (A). (C) HEK293 cells were transiently transfected with the pcDNA3, pcDNA3-E2F1, pU6 or pU6-E2F1 shRNA expression plasmid. 48 hours following transfection, cell lysates were prepared for Western blot analysis using antibodies against E2F1, CD2AP and GAPDH, respectively. Western blots showed levels of E2F1 and CD2AP protein in the cells transfected with specific plasmids. GAPDH was used as a loading control.

In order to observe whether E2F1 affects the expression of CD2AP, the mRNA and protein levels of CD2AP were analyzed in HEK293 cells by RT-PCR and Western blotting. Forty eight hours post-transfection, total RNA was prepared from cells pretreated. Equal amounts of total RNA were used to perform semi-quantitative RT-PCR with primer pairs specific to CD2AP and GAPDH (control). Transient expression of E2F1 enhanced CD2AP mRNA expression compared to vector control (Figure 2A). In contrast, the use of shRNA against E2F1 down-regulated the levels of mRNA (Figure 2B). To confirm the RT-PCR results, cells were pretreated under the same conditions as described above. Seventy-two hours post-transfection, cells were subject to Western blot analysis using anti-CD2AP and anti-GAPDH. As shown in Figure 2C, the CD2AP level exhibited an apparent increase upon E2F1 overexpression. As expected, the use of shRNA against E2F1 down-regulated the levels of the protein secretion of CD2AP. Functionality of the pcDNA3-E2F1 and shRNA directed against E2F1 were confirmed by Western blot analysis: pcDNA3-E2F1 was effective in increasing the E2F1 protein level; pU6-E2F1 shRNA was effective in decreasing the E2F1 protein level (Figure 2C). These results demonstrate that E2F1 can up-regulate the expression of CD2AP at mRNA and protein levels.

### Overexpression of E2F1 transactivates the human CD2AP promoter

**Figure 3:**
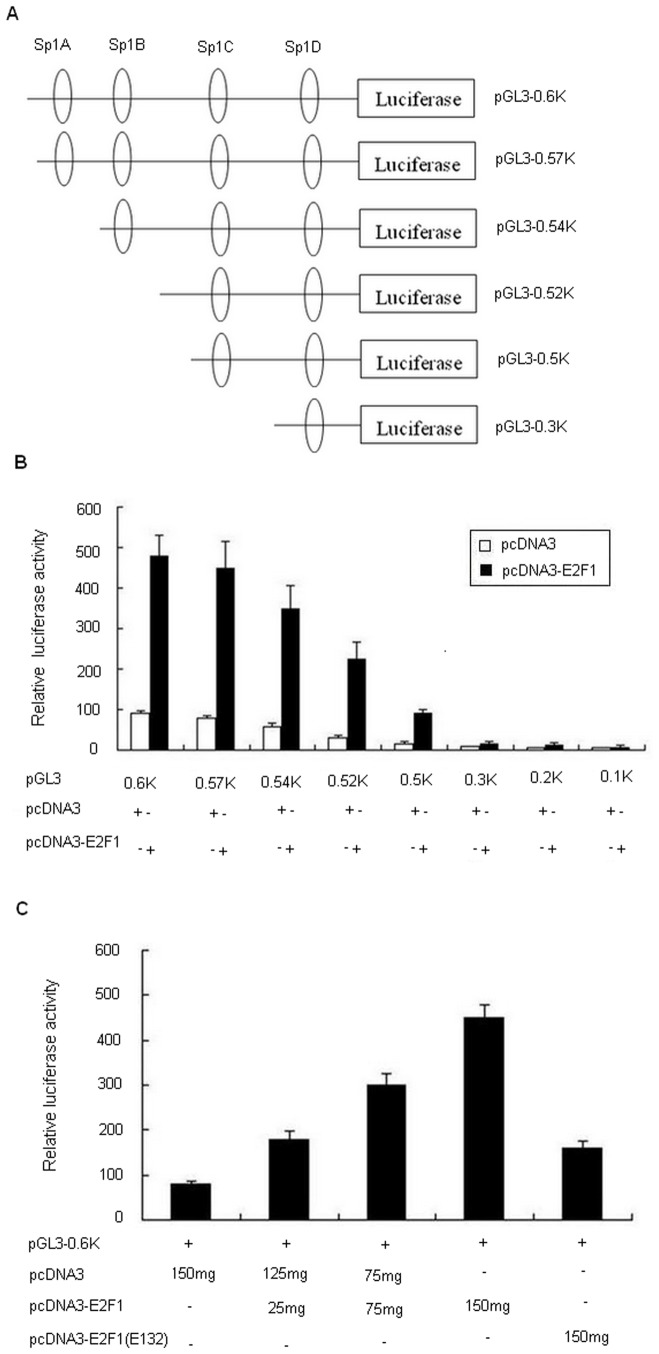
Overexpression of E2F1 up-regulates the human CD2AP promoter. (A) Schematic representation of various the human CD2AP promoter deletions constructs. Putative Sp1 binding sites are indicated. The constructs harboring different fragment of the CD2AP promoter were cloned into pGL3-Basic Reporter Vector (Promega). (B) Determination of minimal promoter region of CD2AP that can be activated by E2F1. HEK293 cells were cotransfected with equal amount (50 ng) of eight CD2AP promoter deletion mutants and the pcDNA3 empty vector (150 ng) or pcDNA3-E2F1 (150 ng). After 24 h, cells were harvested for luciferase assay. Values of luciferase activities are shown as means ± S.D. for three independent experiments, each of which was performed using triplicate samples. Data were normalized to Renilla luciferase activity. (C) Activation of CD2AP promoter by E2F1 is dose-dependent. HEK293 cells were co-transfected with pGL3-0.6k (50 ng), together with either pcDNA3 vector (150 ng), pcDNA3-E2F1 (E132) (150 ng, an E2F1 deletion mutant) or increasing concentrations of pcDNA3-E2F1 (from 25 ng, 75 ng to 150 ng) separately. Luciferase activities were measured and plotted. Data were normalized to Renilla luciferase activity.

To examine whether the human CD2AP promoter can be activated by E2F1, luciferase reporter assays were performed. We first tested the effects of overexpression of E2F1 on human CD2AP promoter activity, and the luciferase reporter assays were performed with a series of CD2AP promoter deletion mutants. The schematic representation of the constructs used in this assay is shown in Figure 3A. Co-expression of E2F1 with a series of luciferase reporter plasmids containing various lengths of the CD2AP 5-flanking regions in HEK293 cells led to 5.3∼6.1-fold increase of the promoter activity compared to that of the pcDNA3 control (Figure 3B). As shown in Figure 3B, all CD2AP promoter fragments containing the four putative Sp1 binding sites displayed significant activation fold under ectopic E2F1 expression. Moreover, as the CD2AP promoter undergoes continuous deduction from 5′ to 3′, the responsiveness to E2F1 did not display significant activation until the Sp1C binding site have been deleted, suggesting that the CD2AP promoter region pGL3-0.5k may represent the CD2AP minimal response element to E2F1. Next, HEK293 cells were cotransfected with pGL3-0.6k, together with either pcDNA3 empty vector, increasing the amount of pcDNA3-E2F1 (25 ng to 150 ng) or pcDNA3-E2F1 (E132) (an E2F1 mutant lacking the ability to bind DNA). As shown in Figure 3C, increasing amount of E2F1 resulted in correspondingly augmented activation fold of CD2AP promoter activity (lanes 2, 3 and 4), whereas the pcDNA3-E2F1 (E132) was ineffective at mediating transcriptional enhancement of the reporter vector (lane 5). These facts indicate that overexpression of E2F1 can transactivate the human CD2AP promoter.

### Knockdown of E2F1 expression by shRNA reduces the human CD2AP promoter activity

**Figure 4:**
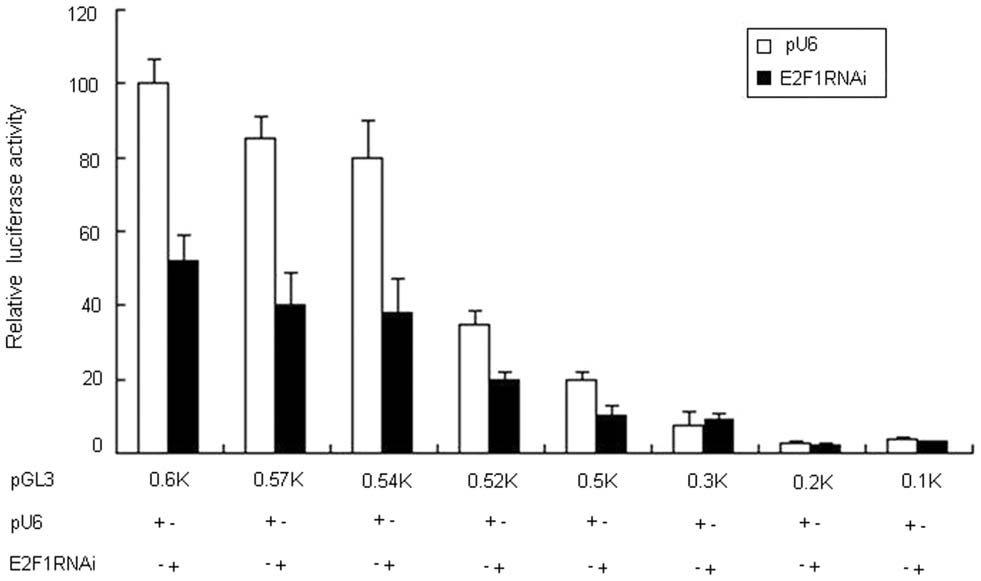
Knockdown of E2F1 expression by shRNA reduces CD2AP promoter activity. HEK293 cells were contransfected with equal amount (50 ng) of eight CD2AP promoter deletion mutants and the pU6 empty vector (150 ng) or pU6-E2F1 shRNA (150 ng). Firefly luciferase activities after 48 h were normalized to coexpressed Renilla luciferase activities. Mean values ± SD of a representative experiment performed in triplicate are shown.

To confirm the role of E2F1 in the regulation of CD2AP promoter activity, we used RNA interference to knock down endogenous E2F1 expression in HEK293 cells. We co-expression of pU6-E2F1 shRNA with pGL3-0.6k, pGL3-0.57k, pGL3-0.54k, pGL3-0.52k and pGL3-0.5k in HEK293 cells individually. This resulted in a 15∼50% reduction of luciferase activity in the presence of pU6-E2F1 shRNA compared to the empty vector pU6. In contrast, no influence on the transcriptional activity of the reporter plasmid pGL3-0.3k, pGL3-0.2k and pGL3-0.1k, which only contains the Sp1D or no Sp1 binding site, was observed (Figure 4). These data further suggest that E2F1 may up-regulate CD2AP promoter activity by binding Sp1A, Sp1B or Sp1C. On the basis of these results, we conclude that E2F1 plays an important role in modulating the CD2AP promoter activity.

### Overexpression of E2F1 changes the DNA-binding activity of E2F1 and Sp1 to Sp1 binding sites without altering the protein level of Sp1

**Figure 5:**
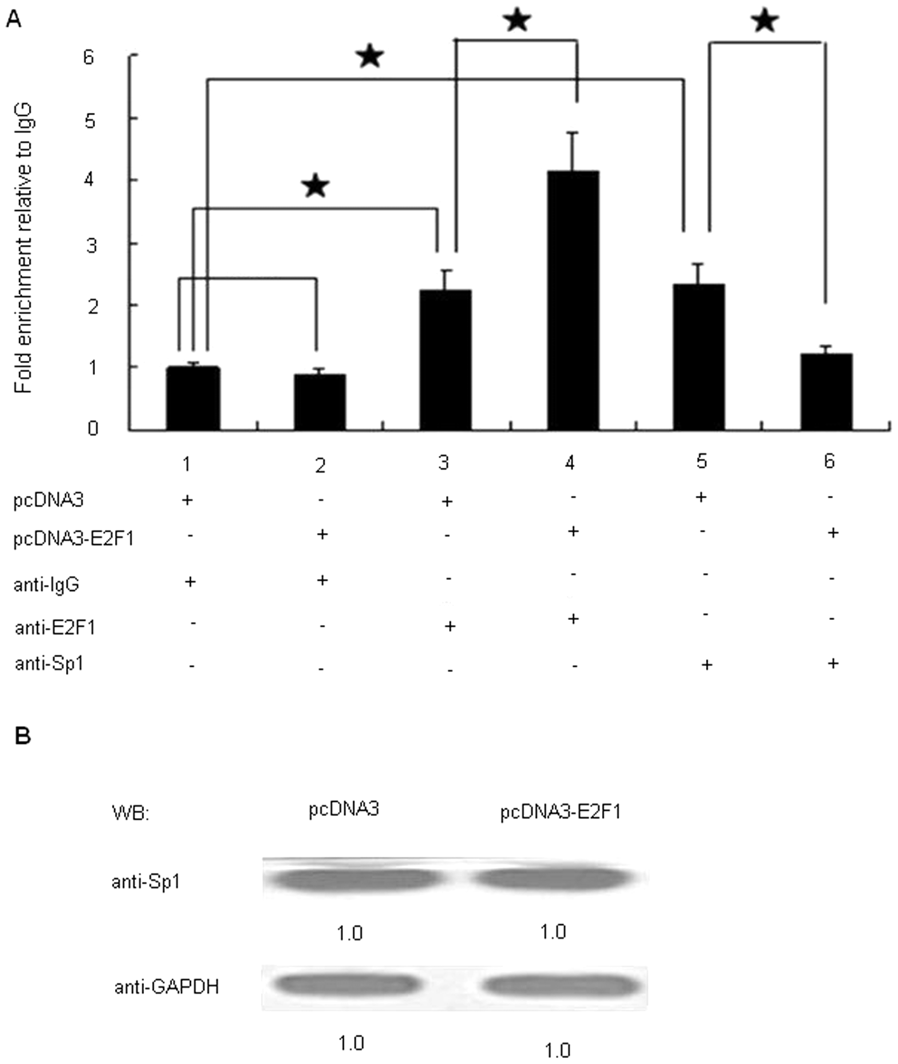
Overexpression of E2F1 changes the DNA-binding activity of E2F1 and Sp1 to Sp1 sites without altering the protein level of Sp1. HEK293 cells were transfected with pcDNA3 or pcDNA3-E2F1 expression vector. (A) 24 h post-transfection, cells were harvested and subjected to ChIP assay. ChIP products were amplified by PCR reaction. Cross-linked chromatin samples from pcDNA3-transfected HEK293 cells, pcDNA3-E2F1-transfected HEK293 cells were respectively immunoprecipitated with anti-E2F1 and anti-Sp1 antibody, and normal rabbit IgG (negative control). Purified DNA was analyzed by PCR using primers as described in Materials and methods. A sample representing 10% of the chromatin used as a starting material for the immunoprecipitation is also shown (Input). The amplified PCR fragments were analyzed on 2.0% agarose gel. Each experiment was performed twice with similar results, and one of them is shown. Data represent the mean ± S.D. Significant differences in E2F1 or Sp1 enrichment of chromatin in pcDNA3-E2F1-transfected cells versus pcDNA3-transfected cells (p<0.05) are labeled with an asterisk (t test). (B) 72 h post-transfection, cells were harvested and subjected to Western blot analysis. Equivalent amounts of total cell proteins were fractionated by SDS-PAGE and probed with antibodies specific for Sp1 and GAPDH. Detection was achieved by chemiluminescence.

To identify whether overexpression of E2F1 changes the DNA-binding activity of E2F1 and Sp1 to Sp1 binding sites, we performed another chromatin immunoprecipitation assay. HEK293 cells were transfected with pcDNA3-E2F1 or pcDNA3 prior to ChIP assay. The chromatin was immunoprecipitated with anti-IgG, anti-Sp1 and anti-E2F1 antibodies and the DNA precipitated in the complexes was subjected to PCR amplification with primers flanking the region containing the four Sp1 binding sites. As shown in Figure 5, over-expression of exogenous E2F1 in HEK293 cells led to a 1.8-fold increase of the relative binding rate of E2F1 to promoter compared to that of the pcDNA3 control. In order to examine the possible role of the Sp1 binding sites in E2F1-mediated activation of the CD2AP promoter, we analyzed whether binding of E2F1 interferes with DNA-protein interaction of the transcriptional factor Sp1. The binding of Sp1 to the CD2AP promoter was also detected in HEK293 cells transfected with either the pcDNA3-E2F1 or pcDNA3 control; moreover we observed a clearly reduced binding of Sp1 in pcDNA3-E2F1-transfected cells than that in the pcDNA3-transfected cells. As showed in Figure 5A, the binding of Sp1 to the CD2AP promoter was 45% reduced by over-expression of E2F1. Collectively, these results indicate that E2F1 overexpression exhibites its activity effect on CD2AP promoter by interaction with the Sp1 binding sites through increasing the binding of E2F1 and reducing the binding of Sp1. In order to observe whether expression level of the Sp1 was impacted by E2F1, we further analyzed it by Western blotting. We found overexpression of E2F1 did not significantly influence the Sp1 on the protein level (Figure 5B). These data also show that overexpression of E2F1 is associated with decreasing the binding of Sp1 and increasing the binding of E2F1 to Sp1 binding sites, whereas the protein level of Sp1 remains unaffected.

### Site-directed mutagenesis of the CD2AP promoter

**Figure 6:**
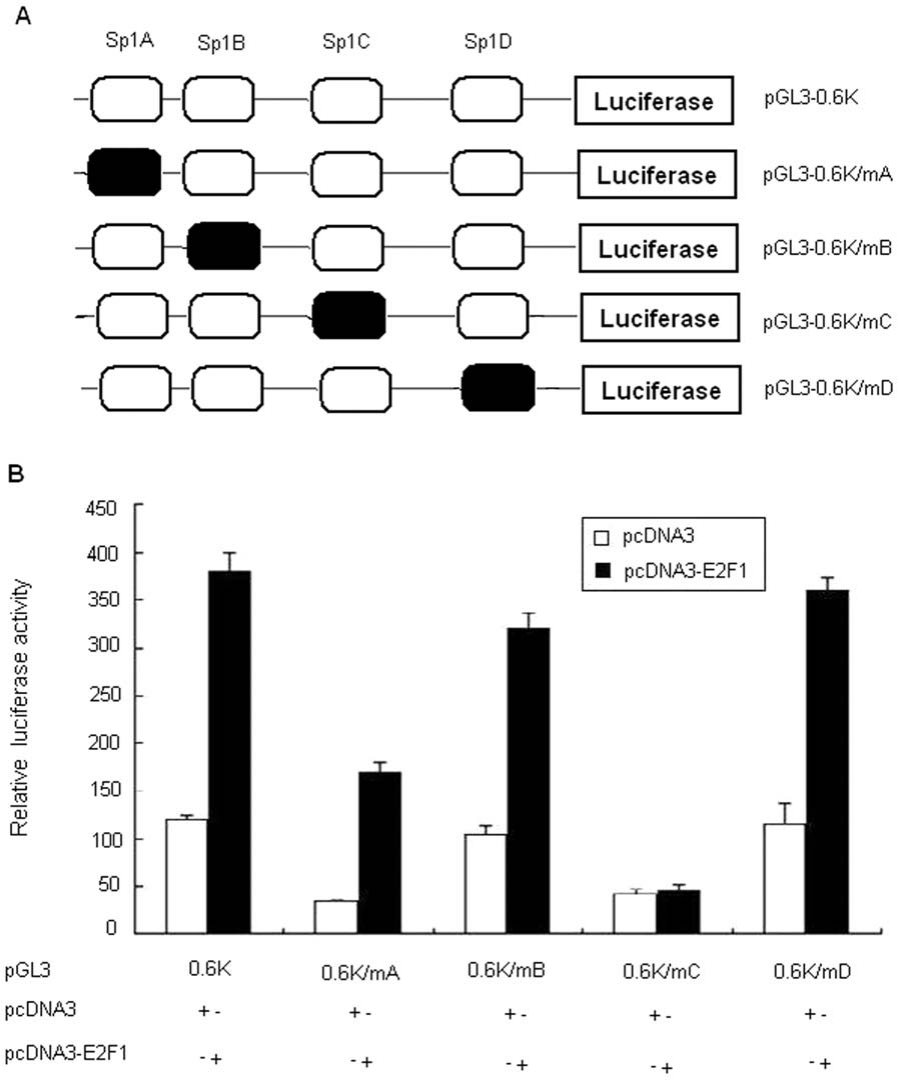
Site-directed mutagenesis of the CD2AP promoter. Mutational analysis of Sp1A, B, C and D, and the effects of co-expression of E2F1 on the promoter activity of CD2AP. Point mutations to disrupt Sp1 binding were made in pGL3-0.6k. (A) Schematic diagrams of Sp1 deletion mutants of CD2AP promoter. (B) HEK293 cells were transfected with either reporter plasmids pGL3-0.6k (50 ng) or one of pGL3-0.6k deletion mutants (pGL3-0.6k/mA, pGL3-0.6k/mB, pGL3-0.6k/mC and pGL3-0.6k/mD, 50 ng) along with pcDNA3 expression vectors (150 ng) or pcDNA3-E2F1 (150 ng). Dates are normalized to Renilla luciferase activity and shown as means ± SD for three independent experiments, with each experiment carried out in triplicate. Fold activation values were measured relative to the levels of Renilla activity.

To further confirm that Sp1 binding sites are responsible for the E2F1-mediated activation of the CD2AP promoter, we generated four mutant promoter constructs with deletion of anyone of Sp1 binding sites respectively. Point mutations to disrupt Sp1 bindings were made in pGL3-0.6k. The sequences containing Sp1A, B, C and D sites were point mutated (Figure 6A). As shown in Figure 6B, Sp1A and C mutations resulted in a 70% and 65% reduction of the transcriptional activity compared to the unmodified promoter respectively, whereas Sp1B and D mutation failed to decrease the transcriptional activity. Overexpression of E2F1 in HEK293 cells by transient transfection up-regulated the promoter activity of pGL3-0.6k/mA, pGL3-0.6k/mB and pGL3-0.6k/mD. However, co-transfection of E2F1 expression plasmid did not up-regulate the pGL3-0.6k/mC promoter activity. These results suggest that E2F1 up-regulates the CD2AP promoter activity by binding to the Sp1C binding site.

**Figure 7:**
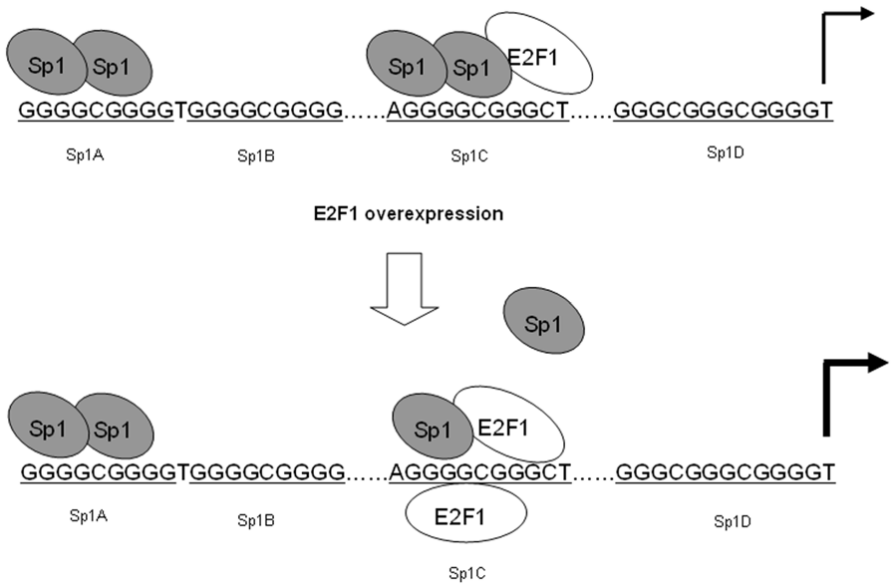
Model of E2F1-inducible changes in transcription factor occupancy of the CD2AP promoter. In untreated cells, Sp1 proteins are in contact with the Sp1A and Sp1C sites in the CD2AP promoter. In cells co-transfection of E2F1 expression plasmid, E2F1 binds to Sp1C site, resulting in the displacement of transcription factor binding to these regions, thereby acting as a transcriptional activator.

In this study, we report a new trans-factor of CD2AP gene in HEK293 cells. Our findings in this study firstly demonstrate that E2F1 can up-regulate the mRNA and protein levels of the human CD2AP in HEK293 cells by the activation of the human CD2AP promoter via binding to the Sp1 binding site. As showed in Figure 7, binding of E2F1 to the Sp1 site would abolish the interaction of Sp1 with the promoter. The increase of the binding of E2F1 to promoter could up-regulate the human CD2AP promoter activity. In summary, our findings may shed new light on the relationship between transcription factor E2F1 and CD2AP. Recent evidences suggest that CD2AP may serve as an important adaptor molecule in preventing podocyte injury and apoptosis, which is involved in the antiapoptotic TβRI/CD2AP/PI3K/AKT signaling module [31]. Future experiments will be aimed at revealing the possible role of E2F1 in anti-apoptosis of podocyte considering that exogenous E2F1 strongly increases the CD2AP transcription as shown in this study.

## References

[1] Dustin ML, Olszowy MW, Holdorf AD, Li J, Bromley S, et al (1998) A novel adaptor protein orchestrates receptor patterning and cytoskeletal polarity in T-cell contacts. Cell 94: 667–677. 9741631 10.1016/s0092-8674(00)81608-6

[2] Weber S, Gribouval O, Esquivel EL, Morinière V, Tête MJ, et al (2004) NPHS2 mutation analysis shows genetic heterogeneity of steroid-resistant nephrotic syndrome and low post-transplant recurrence. Kidney Int 66: 571–579. 15253708 10.1111/j.1523-1755.2004.00776.x

[3] Shih NY, Li J, Karpitskii V, Nguyen A, Dustin ML, et al (1999) Congenital nephrotic syndrome in mice lacking CD2-associated protein. Science 286: 312–315. 10514378 10.1126/science.286.5438.312

[4] Huber TB, Hartleben B, Kim J, Schmidts M, Schermer B, et al (2003) Nephrin and CD2AP associate with phosphoinositide 3-OH kinase and stimulate AKT-dependent signaling. Mol Cell Biol 23: 4917–4928. 12832477 10.1128/MCB.23.14.4917-4928.2003 PMC162232

[5] Schiffer M, Mundel P, Shaw AS, Böttinger EP (2004) A novel role for the adaptor molecule CD2-associated protein in transforming growth factor-beta-induced apoptosis. J Biol Chem 279: 37004–37012. 15213232 10.1074/jbc.M403534200

[6] Cormont M, Metón I, Mari M, Monzo P, Keslair F, et al (2003) CD2AP/CMS regulates endosome morphology and traffic to the degradative pathway through its interaction with Rab4 and c-Cbl. Traffic 4: 97–112. 12559036 10.1034/j.1600-0854.2003.40205.x

[7] Lynch DK, Winata SC, Lyons RJ, Hughes WE, Lehrbach GM, et al (2003) A Cortactin-CD2-associated protein (CD2AP) complex provides a novel link between epidermal growth factor receptor endocytosis and the actin cytoskeleton. J Biol Chem 278: 21805–21813. 12672817 10.1074/jbc.M211407200

[8] Kobayashi S, Sawano A, Nojima Y, Shibuya M, Maru Y (2004) The c-Cbl/CD2AP complex regulates VEGF-induced endocytosis and degradation of Flt-1 (VEGFR-1). FASEB J 18: 929–931. 15001553 10.1096/fj.03-0767fje

[9] Löwik MM, Groenen PJ, Pronk I, Lilien MR, Goldschmeding R, et al (2007) Focal segmental glomerulosclerosis in a patient homozygous for a CD2AP mutation. Kidney Int 72: 1198–1203. 17713465 10.1038/sj.ki.5002469

[10] Smoyer WE, Mundel P (1998) Regulation of podocyte structure during the development of nephrotic syndrome. J Mol Med 76: 172–183. 9535550 10.1007/s001090050206

[11] Gubler MC (2003) Podocyte differentiation and hereditary proteinuria/nephrotic syndromes. J Am Soc Nephrol 14: S22–26. 12761234 10.1097/01.asn.0000067648.75923.68

[12] Shih NY, Li J, Cotran R, Mundel P, Miner JH, et al (2001) CD2AP localizes to the slit diaphragm and binds to nephrin via a novel C-terminal domain. Am J Pathol 159: 2303–2308. 11733379 10.1016/S0002-9440(10)63080-5 PMC1850607

[13] Lu C, Ren W, Su XM, Chen JQ, Wu SH, et al (2009) EGF-recruited JunD/c-fos complexes activate CD2AP gene promoter and suppress apoptosis in renal tubular epithelial cells. Gene 433: 56–64. 19095050 10.1016/j.gene.2008.11.015

[14] Helin K (1998) Regulation of cell proliferation by the E2F transcription factors. Curr Opin Genet Dev 8: 28–35. 9529602 10.1016/s0959-437x(98)80058-0

[15] Trimarchi JM, Lees JA (2002) Sibling rivalry in the E2F family. Nat Rev Mol Cell Biol 3: 11–20. 11823794 10.1038/nrm714

[16] Trimarchi JM, Fairchild B, Wen J, Lees JA (2001) The E2F6 transcription factor is a component of the mammalian Bmi1-containing polycomb complex. Proc Natl Acad Sci 98: 1519–1524. 11171983 10.1073/pnas.041597698 PMC29289

[17] Dimova DK, Dyson NJ (2005) The E2F transcriptional network: old acquaintances with new faces. Oncogene 24: 2810–2826. 15838517 10.1038/sj.onc.1208612

[18] Dyson N (1998) The regulation of E2F by pRB-family proteins. Genes Dev 12: 2245–2262. 9694791 10.1101/gad.12.15.2245

[19] Cam H, Dynlacht BD (2003) Emerging roles for E2F: beyond the G1/S transition and DNA replication. Cancer Cell 3: 311–316. 12726857 10.1016/s1535-6108(03)00080-1

[20] DeGregori J, Johnson DG (2006) Distinct and Overlapping Roles for E2F Family Members in Transcription, Proliferation and Apoptosis. Curr Mol Med 6: 739–748. 17100600 10.2174/1566524010606070739

[21] Bukur J, Herrmann F, Handke D, Recktenwald C, Seliger B (2010) Identification of E2F1 as an important transcription factor for the regulation of tapasin expression. J Biol Chem 285: 30419–30426. 20663889 10.1074/jbc.M109.094284 PMC2945534

[22] Zhou D, Masri S, Ye JJ, Chen S (2005) Transcriptional regulation of the mouse PNRC2 promoter by the nuclear factor Y (NFY) and E2F1. Gene 361: 89–100. 16181749 10.1016/j.gene.2005.07.012

[23] Racek T, Buhlmann S, Rüst F, Knoll S, Alla V, et al (2008) Transcriptional repression of the prosurvival endoplasmic reticulum chaperone GRP78/BIP by E2F1. J Biol Chem 283: 34305–34314. 18840615 10.1074/jbc.M803925200 PMC2662239

[24] Zhang HJ, Li WJ, Yang SY, Li SY, Ni JH, et al (2009) 8-Chloro-adenosine-induced E2F1 promotes p14ARF gene activation in H1299 cells through displacing Sp1 from multiple overlapping E2F1/Sp1 sites. J Cell Biochem 106: 464–472. 19115249 10.1002/jcb.22033

[25] Su XM, Ren W, Lu C, Chen JQ, Wu SH, et al (2009) Functional characterization of the regulatory region of human CD2-associated protein promoter in HEK 293 cells. Am J Nephrol 29: 203–212. 18791326 10.1159/000155658

[26] Lu C, Ren W, Su XM, Chen JQ, Wu SH, et al (2008) CREB and Sp1 regulate the human CD2AP gene promoter activity in renal tubular epithelial cells. Archives of Biochemistry and Biophysics 474: 143–149. 18396147 10.1016/j.abb.2008.03.031

[27] Xu HG, Ren W, Zou L, Wang Y, Jin R, et al (2012) Transcriptional control of human CD2AP expression: the role of Sp1 and Sp3. Mol Biol Rep 39: 1479–1486. 21604172 10.1007/s11033-011-0885-0

[28] Ma Y, Cress WD, Haura EB (2003) Flavopiridol-induced apoptosis is mediated through up-regulation of E2F1 and repression of Mcl-1. Mol Cancer Ther 2: 73–81. 12533675

[29] Xu HG, Ren W, Lu C, Zhou GP (2010) Characterization of the human IRF-3 promoter and its regulation by the transcription factor E2F1. Mol Biol Rep 37: 3073–3080. 19826915 10.1007/s11033-009-9880-0

[30] Xu HG, Ren W, Zou L, Wang Y, Jin R, et al (2011) Direct repression of the human IRF-3 promoter by E2F1. Immunogenetics 63: 189–196. 21225257 10.1007/s00251-010-0505-5

[31] Xavier S, Niranjan T, Krick S, Zhang T, Ju W, et al (2009) TbetaRI independently activates Smad- and CD2AP-dependent pathways in podocytes. J Am Soc Nephrol 20: 2127–2137. 19679673 10.1681/ASN.2008070806 PMC2754100

